# Using a Geolocation Social Networking Application to Calculate the Population Density of Sex-Seeking Gay Men for Research and Prevention Services

**DOI:** 10.2196/jmir.3523

**Published:** 2014-11-18

**Authors:** Kevin P Delaney, Michael R Kramer, Lance A Waller, W Dana Flanders, Patrick S Sullivan

**Affiliations:** ^1^Department of EpidemiologyLaney Graduate SchoolEmory UniversityAtlanta, GAUnited States; ^2^Department of Biostatistics and BioinformaticsRollins School of Public HealthEmory UniversityAtlanta, GAUnited States

**Keywords:** Internet, HIV, MSM, sampling, location services

## Abstract

**Background:**

In the United States, human immunodeficiency virus/acquired immunodeficiency syndrome (HIV/AIDS) continues to have a heavy impact on men who have sex with men (MSM). Among MSM, black men under the age of 30 are at the most risk for being diagnosed with HIV. The US National HIV/AIDS strategy recommends intensifying efforts in communities that are most heavily impacted; to do so requires new methods for identifying and targeting prevention resources to young MSM, especially young MSM of color.

**Objective:**

We piloted a methodology for using the geolocation features of social and sexual networking applications as a novel approach to calculating the local population density of sex-seeking MSM and to use self-reported age and race from profile postings to highlight areas with a high density of minority and young minority MSM in Atlanta, Georgia.

**Methods:**

We collected data from a geographically systematic sample of points in Atlanta. We used a sexual network mobile phone app and collected application profile data, including age, race, and distance from each point, for either the 50 closest users or for all users within a 2-mile radius of sampled points. From these data, we developed estimates of the spatial density of application users in the entire city, stratified by race. We then compared the ratios and differences between the spatial densities of black and white users and developed an indicator of areas with the highest density of users of each race.

**Results:**

We collected data from 2666 profiles at 79 sampled points covering 883 square miles; overlapping circles of data included the entire 132.4 square miles in Atlanta. Of the 2666 men whose profiles were observed, 1563 (58.63%) were white, 810 (30.38%) were black, 146 (5.48%) were another race, and 147 (5.51%) did not report a race in their profile. The mean age was 31.5 years, with 591 (22.17%) between the ages of 18-25, and 496 (18.60%) between the ages of 26-30. The mean spatial density of observed profiles was 33 per square mile, but the distribution of profiles observed across the 79 sampled points was highly skewed (median 17, range 1-208). Ratio, difference, and distribution outlier measures all provided similar information, highlighting areas with higher densities of minority and young minority MSM.

**Conclusions:**

Using a limited number of sampled points, we developed a geospatial density map of MSM using a social-networking sex-seeking app. This approach provides a simple method to describe the density of specific MSM subpopulations (users of a particular app) for future HIV behavioral surveillance and allow targeting of prevention resources such as HIV testing to populations and areas of highest need.

## Introduction

In the United States, human immunodeficiency virus (HIV) continues to have a heavy impact on men who report having sex with men (MSM) [1,2]. Although HIV incidence is increasing among MSM overall, there are pronounced disparities in both prevalence and incidence in the United States within the MSM HIV epidemic by race/ethnicity. A Centers for Disease Control and Prevention surveillance study conducted in 2008 [3] found that black non-Hispanic MSM were significantly more likely to be living with HIV than were white non-Hispanic MSM (28% vs 18%), and among those living with HIV, blacks were also significantly more likely to be unaware of their HIV infection (59% vs 26%). The disparity in HIV prevalence is consistent with a marked difference in estimated incidence of new infections for young minority MSM. From 2006-2009, black MSM under age 30 experienced a 47% increase in the estimated annual number of new infections and in 2009, there were more new infections in black MSM under age 30 than in white MSM under age 39 and more than all Hispanic MSM [4].

As a result, there is renewed emphasis [5] on identifying reasons for these disparities [6,7] and developing and providing interventions specifically for young minority MSM. However, the number of HIV prevention interventions implemented and evaluated with young minority MSM remains relatively low [8,9]. One reason for the lack of interventions specifically targeted to black MSM may be difficulty identifying a sampling frame for this population [6,8]. Stigma experienced by black MSM [10-12] may pose particular challenges in enumerating and accessing these men for provision of services [11]. A variety of sampling methods have been developed to access hidden or marginalized populations [13-18], with varying degrees of success [17-24].

Social networking websites and apps represent novel means for individual communication. A variety of new social networking tools designed for MSM are now available for most smartphones [25-28], and combined, these apps have more than 6 million users and 10,000 new users added daily. Many of these apps build their services on the ability to use the geolocation features available on most phones and other communication devices (iPods, iPads, and tablets) to provide location information for other app users, including their geographic proximity (in feet or miles) to the user’s location. In this paper, we describe a methodology for using the geolocation features of one of these apps as a novel approach to calculating the population density of men using the app at given times and describe how to use this density measure to highlight areas with a high-density of minority and young minority MSM.

## Methods

### Overview

**Figure 1:**
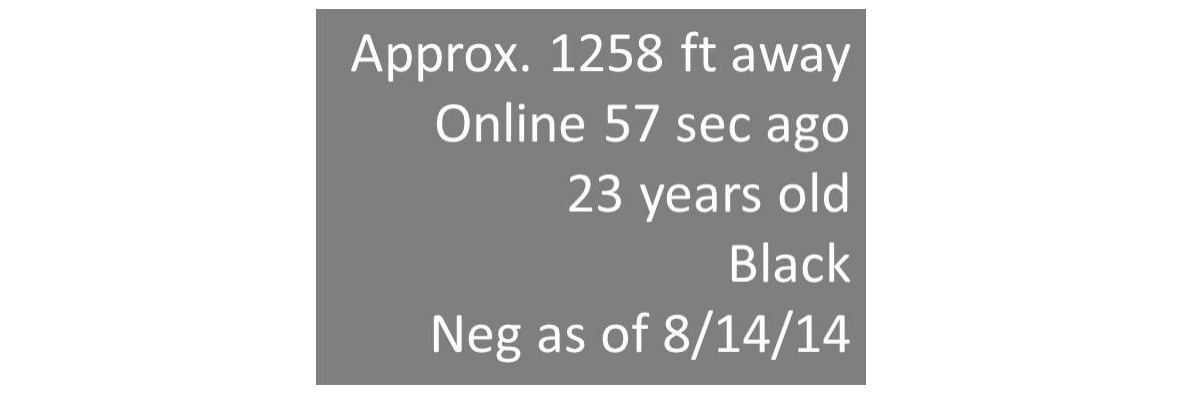
Hypothetical example of social networking application profile data provided by application users (we used age, race, and distance in feet from our location in this analysis).

**Figure 2:**
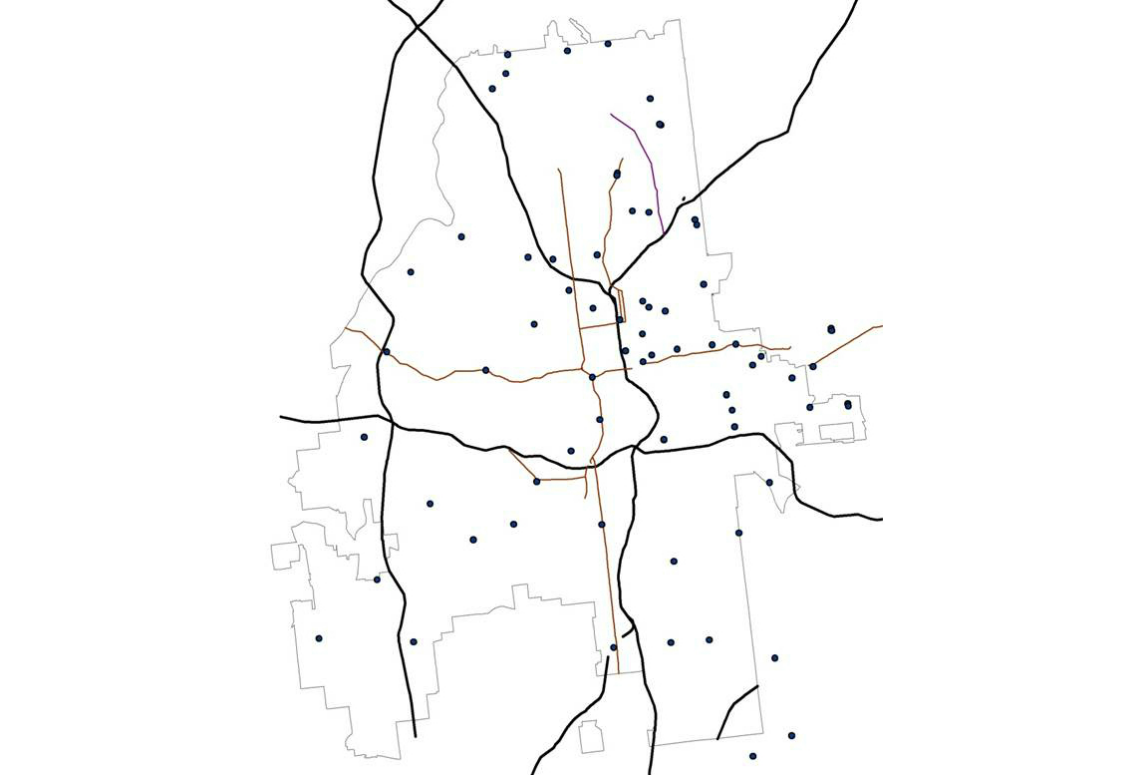
Map of Atlanta (gray outline) including major interstates (black lines) and selected major roads (dark red lines); points in the figure represent 79 data collection locations.

To pilot the study methodology, we chose a sexual networking app and collected data from publicly available profiles at sampled locations around the city of Atlanta, Georgia. App profiles (see Figure 1 for a hypothetical example showing the types of data typically included in user profiles) include information on the linear distance from the user to each other member, in feet for distances less than one mile, and miles for larger distances. For example, the person whose profile is represented in the first panel in Figure 1 was 1258 feet from our sampling location when the profile was viewed. Although we piloted this approach with several of the available apps [25-28], data generated for this paper were from a single app whose name is not revealed at the request of the developer. App profiles indicate the distance but not the direction of the person in question. In order to develop measures of density of users, we began by establishing a grid over Atlanta and selecting points within the grid at which to collect information (Figure 2). Points were selected systematically with the following protocol: we selected a starting point near one author’s (KPD) home and drove along major roads to sample at roughly 2-mile intervals through most of the city. In areas with a high density of app users, we used a sampling strategy designed to collect data more frequently at closer intervals (see below). At each point where profile data were observed, study staff used the “GeoLocation” app [29] to pinpoint the location of data collection to latitude and longitude.

### Validation of Geolocation Data

**Figure 3:**
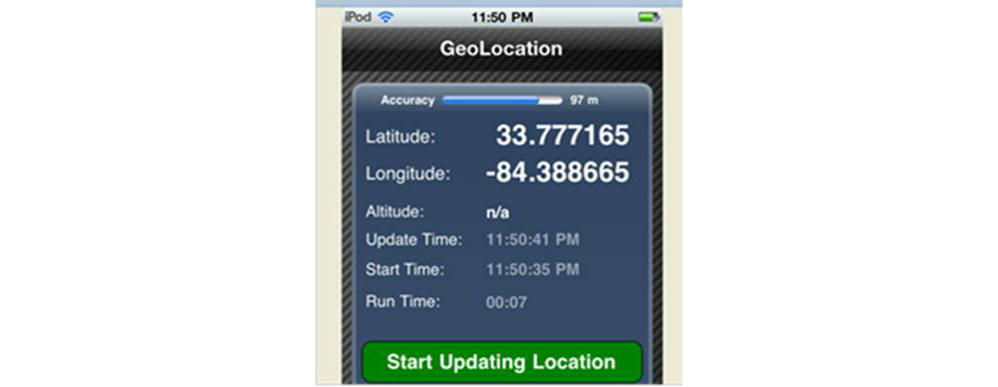
Example of the GeoLocation app used in this study, which relies on cell towers and an Internet connection and provides latitude and longitude in decimal degrees.

In order to assess the accuracy of the geolocating app, we also recorded the global positioning system (GPS) location at a subset of the same points at several different days/times using both GeoLocation and a GPS unit (Garmin model GPSmap 60CS [30]). The GeoLocation app was found to be consistent with the GPS unit, with the mean of the difference between them of 144 feet (range 7-344 feet) over a total of 25 sampled points. The GeoLocation app was also used at the same 10 locations 6 months apart and found to give consistent results with a mean of the difference in location coordinates of 76 feet (range 0-232). Thus we found it sufficient to use the free GeoLocation latitude and longitude data available on the same device as the social networking app for our purposes, rather than using two different devices for data collection. See Figure 3 for a screenshot from the GeoLocation iPhone app, available from [29]. Similar tools available for Android devices [31] were not evaluated in this study.

### Data Collection

At each sampling point, study staff collected screenshots of user profiles. These apps sort profiles based on distance from the user to other users. We collected profile data for either the 50 closest users or for all users within 2 miles of the sampling point, whichever was less. Profiles were saved on a password-protected iPod Touch. These data were entered into a database after field collection of the screenshots. Staff also recorded the day and time of data collection at each point. We calculated the total time spent collecting data as a process measure for this pilot study.

For each profile recorded, we extracted self-reported race and age, and the reported distance from the sampled point (see Figure 1). Race was categorized as “white”, “black”, or “other”, and age was recorded as a continuous variable. If a profile included no information on race or age, this was indicated with a missing value in the database. Because the main objective was to compare the distribution of persons reporting their race as white to those reporting their race as black, when either race or age were missing, we recorded missing race as “other” and missing age as missing. Individual profile data from each sampled point were aggregated as the number of users by self-reported race (grouped as white, black, and other) and self-reported age group (grouped as 18-24, 25-30, >30, or unknown), and summary measures comparing those reporting black or white race in their profiles (further described below) were calculated for Atlanta.

### Sampling Strategy

**Figure 4:**
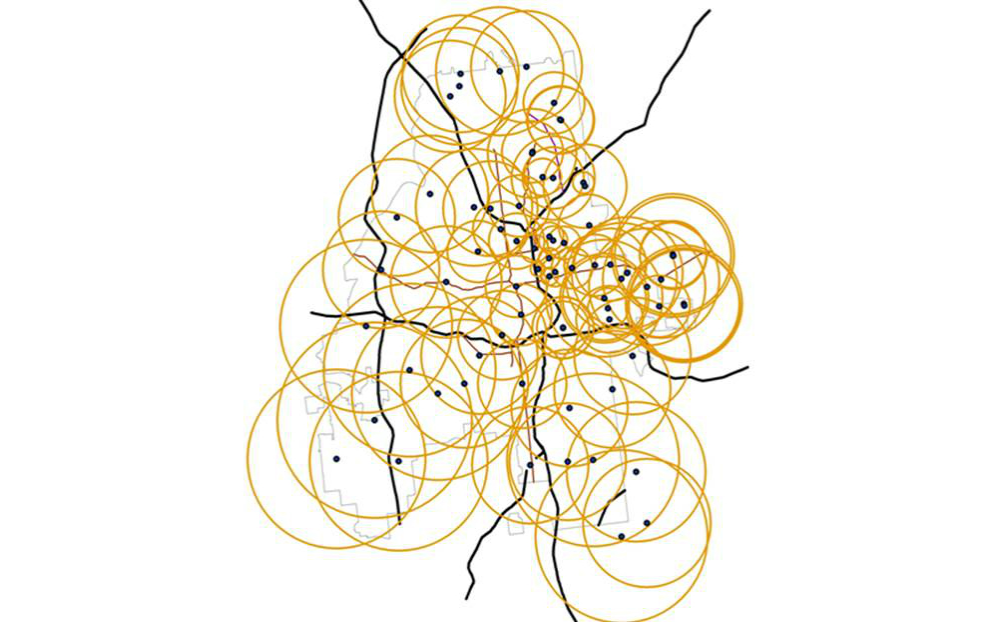
Map of Atlanta showing 79 data collection points from profiles on a sex-seeking networking app; radii of yellow circles represent distance to user sample at the maximum distance from the sample point, and overlapping circles completely cover Atlanta, with smaller circular areas used for data collection where there were the largest numbers of application users.

At points where there were greater than 50 users within less than a 2-mile radius, we recorded the maximum distance to the 50^th^ closest user (ordered by distance) and moved this same distance along city streets to establish the next sample point. Thus smaller radii were used in areas with a higher density of users. Figure 4 shows the sampling radii for each point: the smaller circles represent the areas of Atlanta with the highest density of users, and thus larger numbers of individual profiles available within a given (eg, 2 mile) radius. Because we collected different numbers of users from circles of different radii, we chose to standardize these measures to a common area, for example, converting each observation into the number of users within 1 mile of the point (thus describing a circle with a mile radius and/or an area of π square miles), and stratifying these measures by race and age group.

### Analysis

The data in this study provide a somewhat unique challenge to geospatial statistical methods because they combine the characteristics of point and area processes [32-37]. Data are collected at points on a grid, but the data at that point represent a density over an area of sampling in a concentric circle around that point. Still, the data are more analogous to point data, with the measure collected at each point representing an area rather than an individual data point. Thus we chose to treat these densities of users per square mile as the measure of interest but use point data statistics [32,38,39] to summarize over the entire study area. ArcGIS [39] performs kernel smoothing to estimate the density measured at each sample point where each sample point is weighted by the observed population density at that point. In our case, the Kernel Density smoother [34] counts every white and black user observed at that location. For example, a point at which we observed 12 profiles within 2 miles, including 8 white and 4 black users, would be counted 8 times in the white density measure and 4 times in the black density measure. Next, these weighted values for each point are also averaged with other points within a specified radius [32,36,37], resulting in a smoothed surface representing the density of users, by race, in the sample space. The kernel approach may place non-zero density in areas where no data were collected, but only as a result of averaging between points separated by the area with no data. We also experimented with methods for interpolation of spatial data such as kriging [32,38] and found similar results. We focus on kernel density estimates here. As noted above, sampling was conducted at different times and days of the week over a 6-month period (see Multimedia Appendix 1 for documentation of days and times sampled). While an in-depth analysis of time of day and day of week variability is of interest for future research, to illustrate our approach, we present the kernel densities calculated here as averages over sampled days and times.

After estimating the population density, we used ArcGIS to compute the mean and standard deviation for the calculated density measure over the entire sample space. We compared density surfaces through ratio and difference measures via the Map Algebra tool in ArcGIS, which solves standard algebraic equations at each point in a grid across the density surface and creates a new map displaying the results of these calculations. When comparing the density of users, the difference between surfaces for different races, for example, (density of black users – density of white users) has the property that its null value (no difference) is zero, and if positive, it identifies an area with a higher density of black users than white users. This represents an absolute difference in the densities of the two groups. When positive, this approach identifies areas where it might be easier to recruit black users because the density of black users is greater in absolute terms (ie, the number of excess individuals). We note that this example says nothing about the magnitude (size of the density of black and/or white users), only that one number is bigger than the other. To capture areas where there are relatively more black users than white users (ie, the ratio of black to white users is higher), we also calculated the ratio of the two density surfaces.

As a further exploration of the possibilities with the approach, we also considered a measure to highlight areas with the largest densities for each race and then compare these areas as follows. First, for each density surface (eg, the density of black users <25 years of age) we identified areas with the highest density values (density value > mean + 2 SD). For example, if the estimated mean density for white users was 14/square mile with standard deviation of 7, we would ask ArcGIS to select points with a density of white users greater than 28. We then used Map Algebra to calculate the difference between the surfaces including these highest density points for each race according to the following formula:

I(Density of black users > mean + 2SD of estimated kernel density distribution) − I(Density of white users > mean + 2SD of estimated kernel density distribution)

where I(statement) represents an indicator function with value 1 if the statement is true and zero otherwise. This equation takes only three values: zero when a point is greater than mean + 2SD of both distributions or neither is greater than mean + 2SD; 1 when a point is greater than the mean + 2SD for only the first distribution; and -1 when the point is only greater than the mean + 2SD of the second distribution. This measure identifies not only locations with more users of a given race, but also locations with the highest density areas overall. Similar measures can be constructed to highlight other features of interest, for example, comparing densities by age group or combinations of race and age. Finally, to provide some context to our results, we present them in relation to the location of recruitment sites seeking to enroll MSM for two ongoing HIV prevention studies in Atlanta.

## Results

Over a 2-week period, we spent a total of 21 hours traversing Atlanta, collecting data at the 79 sample points (Figure 2) covering 883 square miles of area (Figure 4) in order to collect overlapping circles of data and cover the entire 132.4 square miles in the city of Atlanta. The average radius of data collection at each sample point was 1.65 miles, with smaller radii resulting from the more densely populated areas.

We extracted profile data (race and age) for 2666 user profiles. Of these, 1563 (58.63%) were white, 810 (30.38%) were black, 146 (5.48%) were some other race, and 147 (5.51%) did not report a race in their profile. The mean age was 31.5 years, with 591 (22.17%) between the ages of 18-25, and 496 (18.60%) between the ages of 26-30. Age was more likely than race to be missing from profile information with 593 (22.24%) of profiles sampled not providing age information. The remaining 37% of profiles reported ages greater than 30; whites were more likely to report being >30 years of age than blacks (46% vs 25%, *P*<.001). Black users were younger than white users (median 28 vs 33 years, *P*<.001 via the Wilcoxon Sign rank test).

**Figure 5:**
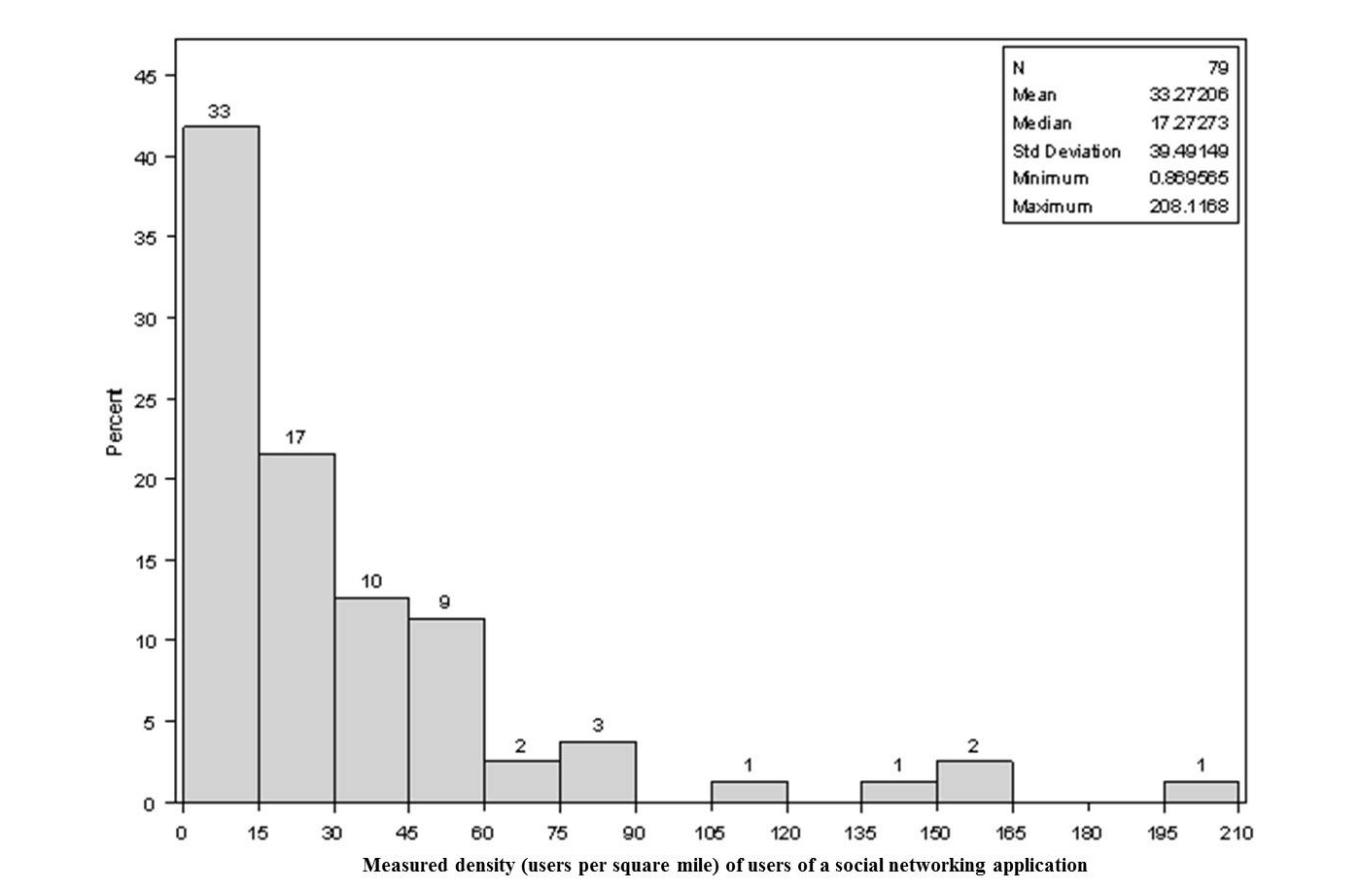
Histogram showing distribution of observed density of social network application users per 1-mile circle for the 79 sampled locations in Atlanta (inset includes statistics for the distribution, which is highly skewed with SD estimated to be larger than the mean; numbers above bars are the number of sample points with density along the X axis and the Y axis representing the percent of all points with this density).

Across the 79 sampled points, the mean number of users was 33 per square mile, but the distribution of users across points was highly skewed with median of 17 and range 0.86-208 (Figure 5).

**Figure 6:**
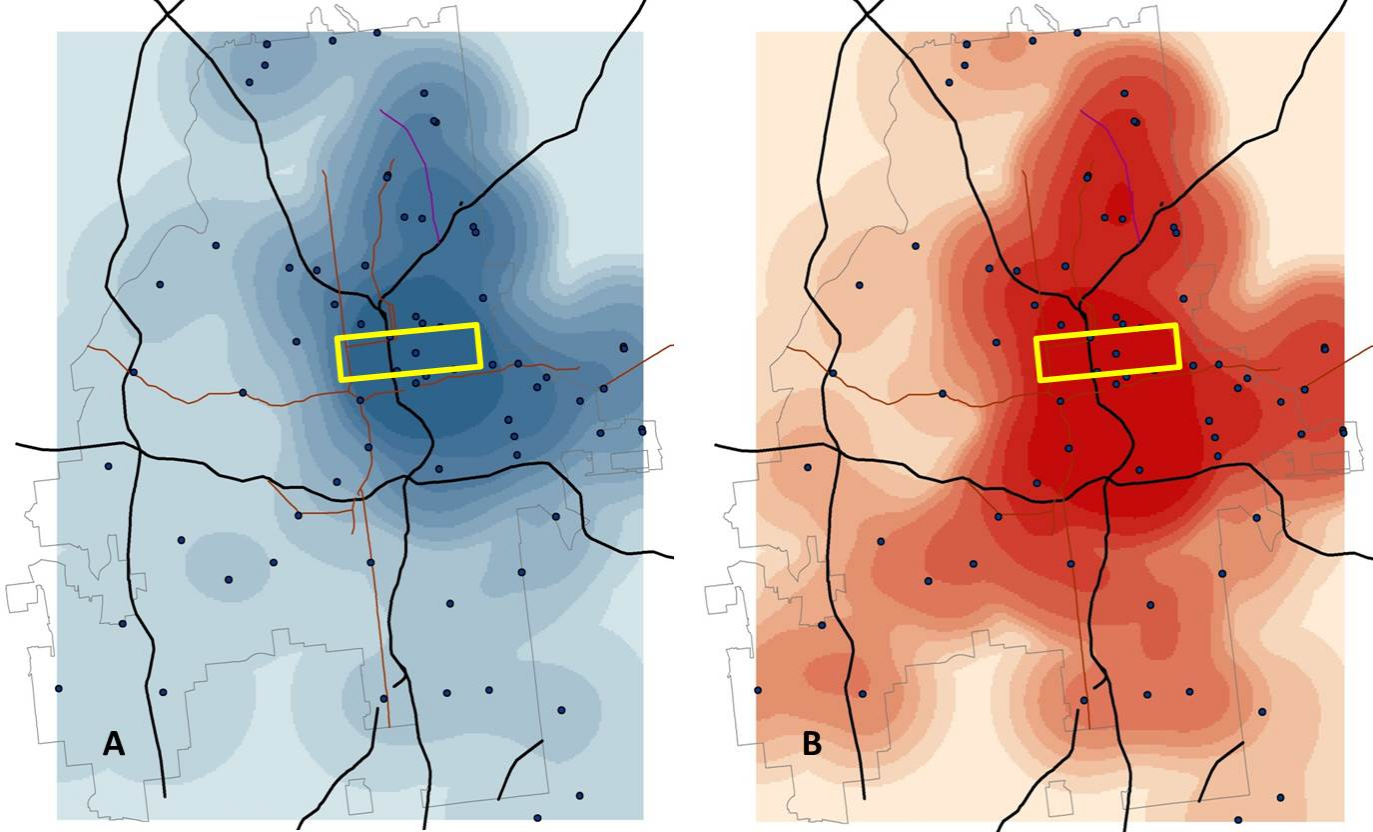
Estimated density of white (A) and black (B) social network application users in Atlanta (gray outline), showing major highways (black lines) and roads (dark red lines) and highlighting the “Midtown” area of Atlanta (yellow rectangle); kernel densities estimated from sample data standardized to 1-mile circular radii and smoothed to 2 miles using a Gaussian smoother that concentrates the majority of the density at the sample point and averages over all adjacent data points within the smoothing radius.

**Figure 7:**
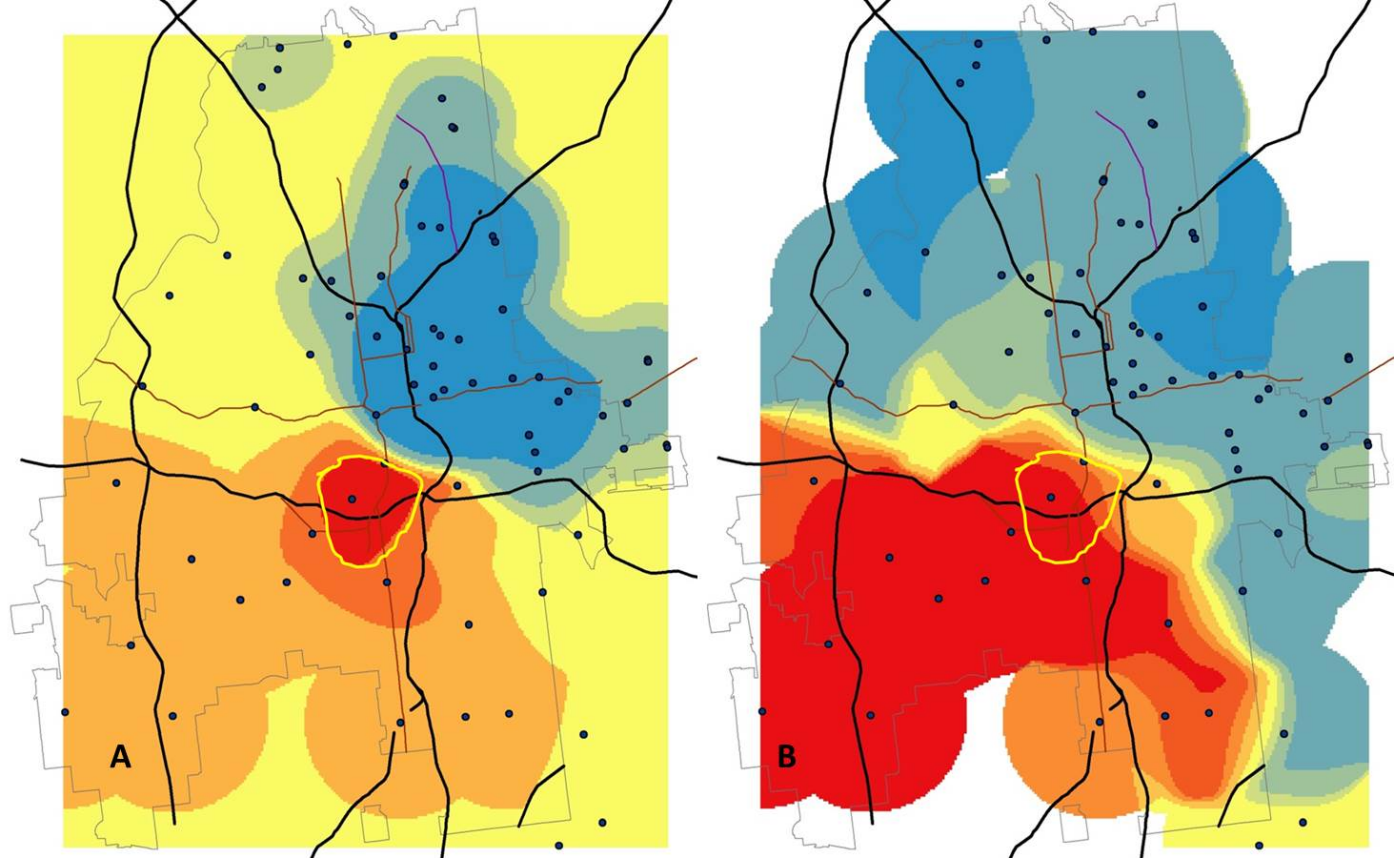
Comparison of the density of black and white social networking application users in Atlanta. Panel A shows the absolute difference in users (Density of black users – Density of white users) color-coded so that areas with more black users appear red and those with more white users appear blue. Yellow regions are areas where the two densities are similar. Panel A highlights a small section of the city (the area shaded the darkest red) where there are many more black than white application users. Panel B shows a comparison of the relative size of the densities of black and white users (Density of black users/Density of white users). With this measure, Atlanta is divided nearly in half, with relatively more black users in the southwest and more white users to the north and east. The yellow band in Panel B shows the region with the highest absolute excess of black users for comparison purposes.

Figure 6 shows the density of app users, smoothed using a kernel density function with a 2-mile radius, for white (A) and black (B) users. A 2-mile radius was chosen as the smoothing parameter because it was the next largest integer that covered the average radius of 1.6 miles in the sampled points and also was the maximum distance to which we sampled data when a sample point had fewer than 50 users. Multimedia Appendix 1 shows the analogs of Figures 6 and 7 with a 1-mile kernel density smoothing parameter for comparison; the results were not qualitatively different. The highest density of white users (the darkest blues in the first panel in Figure 6) concentrates in Midtown Atlanta (roughly bounded by the yellow rectangle on the map). While much of the highest density of black users also concentrates in this area, it is clear that there are areas with high densities of black users further south and to the west (to the lower left) of Midtown. The kernel approach smooths observations according to a two-dimensional distribution centered at the observed point and declining out to the radius used to define the search area, essentially “spreading” observations from sample points across the study area. For example, the density values for white users over the 79 sample points ranged from 0.3 to 154 profiles per square mile, but the range of values for the smoothed density shown in the first panel in Figure 6 was 0-57 profiles per square mile. For the 1-mile smoothed density (Multimedia Appendix 1) the range (0-138) was closer to the observed values, but with many more points with density estimates of zero (ie, observations were not “spread” as far).

There are several ways to compare surfaces to illustrate local differences between the densities of white and black users. Figure 7 shows two similar but nonidentical ways to compare these densities. Panel A in Figure 7 shows the difference between the two surfaces, colored so that areas with higher absolute density of white users are blue and areas with higher density of black users are red. Panel B in Figure 7 shows the relative difference, with areas where the ratio of black to white profile densities is higher than one as red and lower than one as blue.

The ratio measure shows that most of Southwest Atlanta has relatively more black user profiles observed than white profiles, but when we compare the map with that of the overall number of black users, we find a much smaller region in which to focus efforts, that is, south and west of Midtown, shown with a yellow band in Figure 7.

**Figure 8:**
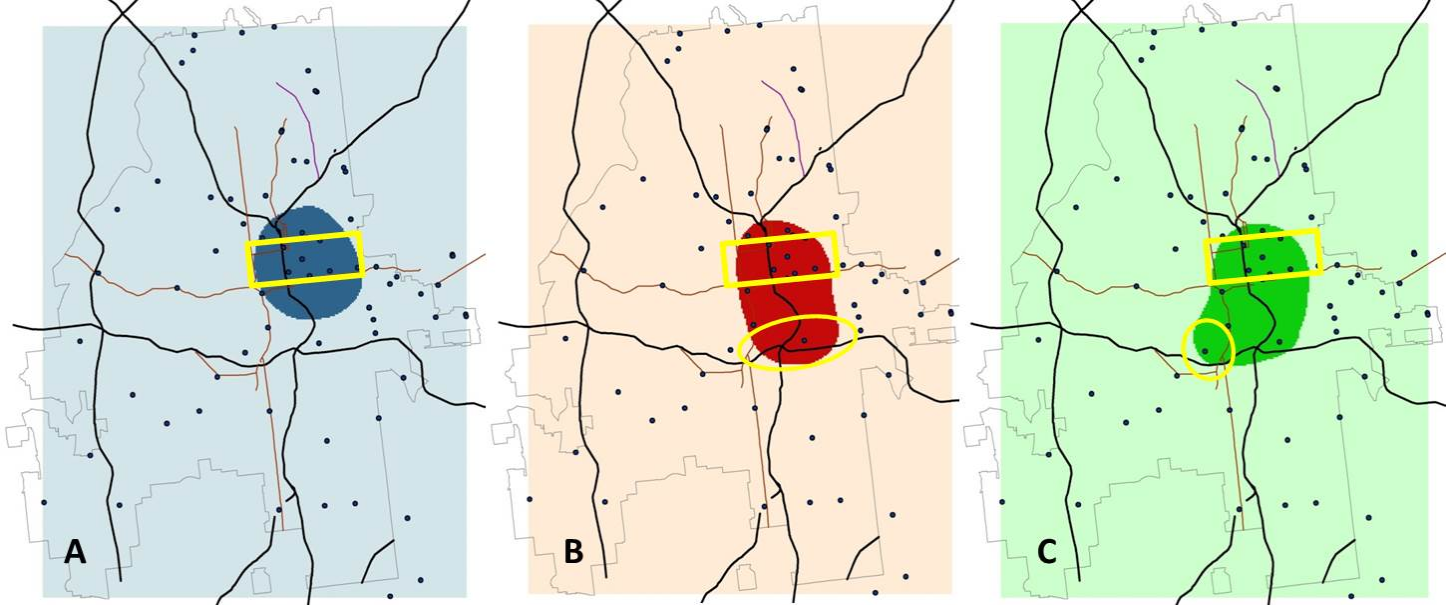
Density of social networking application users in Atlanta, highlighting points with values >95th percentile of estimated kernel densities for white (Panel A), black (Panel B), and young black (<25 years of age, Panel C) users. For Panel A, points with an estimated density >17.2 users/mile^2^ are highlighted dark blue; for Panel B those >5.65/mile^2^ are dark red, and for Panel C >2.8/mile^2^ are dark green. The yellow rectangle highlights the midtown area of Atlanta for reference. The yellow oval in Panel B highlights an area with high density of black users but not white users. The yellow circle in Panel C highlights an area with a high density of young black users, but not black users overall (ie, an area highlighted in Panel C but not Panel B).

A third way to visualize differences between the surfaces is to focus on the areas with extreme values. This provides a within-density comparison: over the entire surface of the density of black user profiles, where is the density the greatest? In Figure 8, we highlight the regions with density greater than the mean+2 standard deviations over the entire map, separately for all white (A), all black (B), and young black (<25 years old, C) users based on data in their observed profiles. This approach again highlights Midtown Atlanta (yellow rectangle) as the region with the most users observed in each graph.

**Figure 9:**
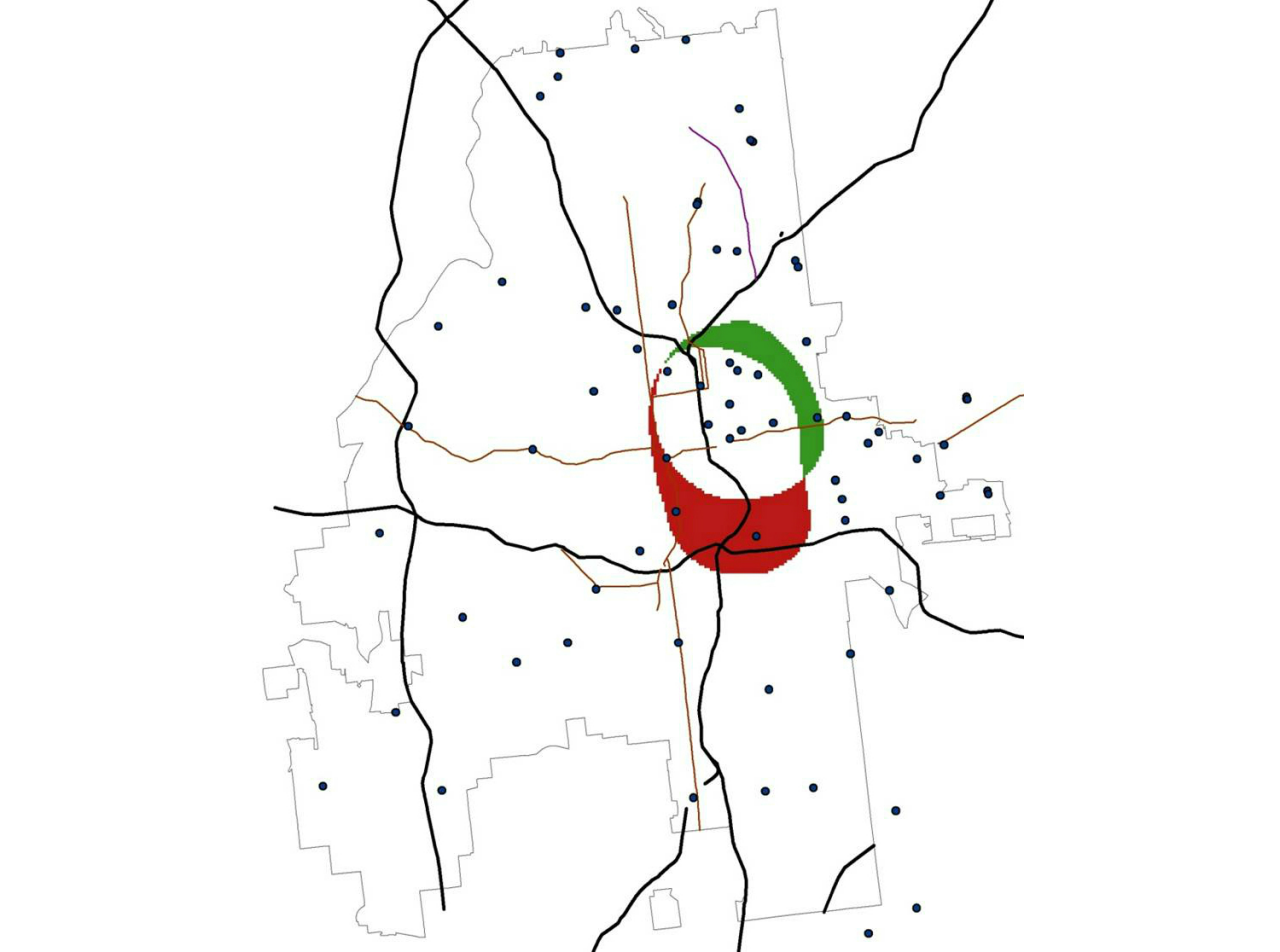
Difference between extreme values of estimated kernel densities of white and black users of a social networking application. We use the formula I(Density of black users > mean+2 standard deviations) – I(Density of white users > mean+2 standard deviations). Figure shows regions where the values of this equation are -1 (green shading, indicating areas with extremes of density for white but not black users), 1 (red shading, indicating areas with extremes of the density for black but not white users), and 0 (white shading indicating areas that are either not extremes of either density or are extremes for both races).

Figure 9 calculates the difference between the first two panels in Figure 8 and shows that black user profiles have high density much further south than white profiles.

The third figure included in Multimedia Appendix 1 compares the difference between the 1-mile smoothed densities for young black and all black users (an analog to Figure 9 but comparing panels B and C of Figure 8). Overall the results are similar, but there are a few additional areas (highlighted in Multimedia Appendix 1 figure) with extreme densities of young black users that did not appear in the 2-mile estimates shown in Figure 8c or 9).

## Discussion

### Principal Findings

We sampled 2666 profiles from a mobile phone–based social networking app at 79 sites in Atlanta and, under our sampling protocol, observed a mean of 33 app users per square mile. We also identified areas where there were more black and young black user profiles observed compared to white user profiles, describing three different summary measures of the density of profiles in a sampling frame. Finally, we showed the impact of the choice of the kernel radius in construction and interpretation of such data.

### Application of the Proposed Methods

**Figure 10:**
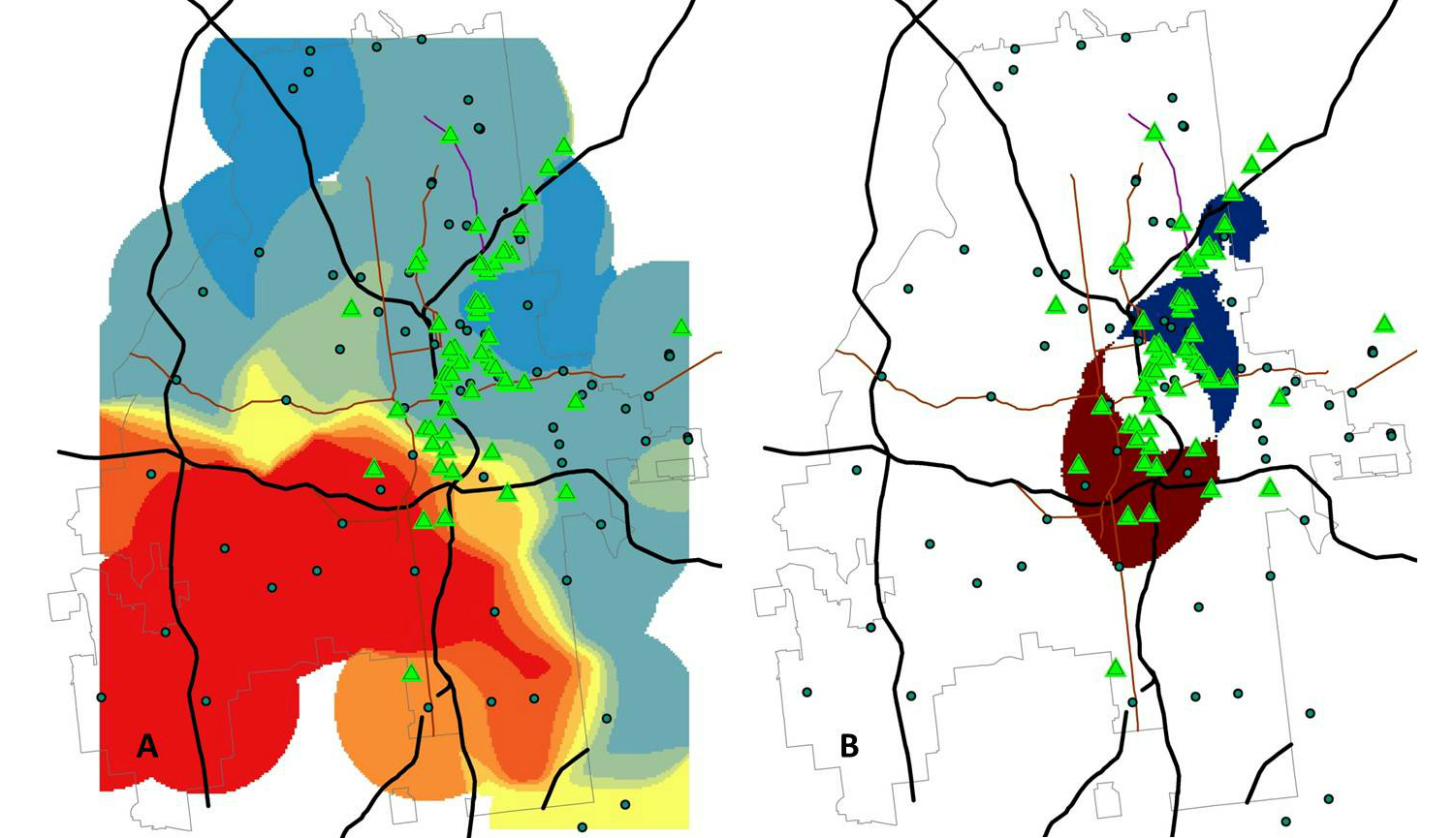
Application of two density metrics to evaluate recruitment for HIV prevention studies in Atlanta, showing recruitment venues currently in use for two HIV prevention studies (green triangles). Panel A shows few recruitment locations in the southwestern part of Atlanta, where there are relatively more young black application users than white users. Panel B uses the formula (Density of young black users > mean+2 standard deviations) – (Density of young white users > mean+2 SD). Regions where the values of this equation are -1 (blue shading, indicating areas with extremes of density for young white but not young black users), 1 (red shading, indicating areas with extremes of the density for young black but not young white users), and 0 (white shading indicating areas that are either not extremes of either density or are extremes for both races) can then be compared to the locations of current recruitment venues.

The goal of this study was primarily descriptive, in that we sought to describe a method for calculating the density of user profiles by race and age in Atlanta, and to compare and contrast the information provided by different outcome measures that can be constructed from these data. In addition, the methods described here may have practical application in HIV prevention research. The results are promising and illustrate how the use of self-reported location data can provide information on the geographic distribution of users in time and space. The study methodology could provide a more efficient way to identify locations for recruitment of MSM in future studies. Significant time and effort is spent on formative research to develop sampling frames for studies of MSM [15,21]. The goal of such formative research is to identify locations for sampling MSM using time space sampling methods [15]. Our methodology, based on the geolocation data incorporated into popular social networking apps, allowed us to quickly describe the density of sex-seeking MSM in Atlanta. Furthermore, we were able to use profile information to stratify these density measures by race and age. This might allow for oversampling or exclusive sampling in areas of the city that are expected to yield a particular subset of the population, for example, young black MSM. As an example, Figure 10 illustrates how these data can inform study implementation in practice. Figure 10 shows Panel B of Figure 7 and a variation of Figure 9, along with recruitment venues currently in use for two HIV prevention studies in Atlanta (green triangles). Panel A in Figure 10 shows that, to date, there have not been very many sampling locations in the southwestern part of Atlanta, where, based on the ratio of the density of black to white app users, there are relatively more black users than white users. However, Panel B in Figure 10 shows the difference between extremes for the densities of young white and young black users of the social networking app, using a formula similar to that used to calculate Figure 9. Looking at this representation of the data, we see that we have identified recruitment venues in an area of the city where there are the most young black app users and not that many white users. In this case, while going further into the areas of higher relative densities of black users might yield additional recruitment sites, we seem to have covered the areas with the highest number of both black and white users. Also, we find that there are not many recruitment sites outside of the area with the highest densities of white users, black users, or both, confirming that past recruitment sites were located in parts of the city where there are the most app users overall. Further potential applications of this methodology include identification of areas with need for prevention services, for example, overlaying HIV testing locations on the density grid to identify local areas with greatest unmet need.

Since the early 2000s, there has been a significant rise in Internet usage by MSM [40-43] and young minorities [44-46]. Three different groups have found that gay men now report meeting the majority of their sex partners online [40,47-49], and many [43,47-49] but not all [50] studies of sex behavior have shown increased reports of behaviors associated with higher HIV risk among partners met online compared to offline. The most popular and well-studied of these location-based social networking apps is Grindr [51-53], which is currently being used by over 4 million men worldwide [25] and is likely to continue to grow in popularity. MSM use this app for a variety of purposes, but a survey of Grindr users in Los Angeles found that 76% have had sex with someone they met on Grindr [51], suggesting that Grindr users are using the app to help find sex partners. Many other similar apps exist such as Adam-4-Adam, Jack’d, and BoyAhoy [26-28], and our methodology can be applied to any such app that provides data on race and age as well as distance to the user within member profiles. In our research, we have found that users of these apps vary by race and less so by age, with, for example, a greater proportion of white men reporting using Grindr and more black men reporting using Jack’d (unpublished Emory University data). In this study, although we illustrate our approach using only one app (and have chosen not to identify the specific app used to generate these data), we did validate the methodology with more than one app. Any of the apps that report race, age, and other characteristics of interest (eg, HIV serostatus), as well as geographic distance from the user’s present location, could be used to make density maps and calculate summary statistics using the methods we report in this paper. In some cases, it may be useful to calculate one or more density measures with more than one app to try to get a better overall picture of the spatial distribution of men using sex-seeking apps in a given location.

Because users of these apps make both their profile information and their location public, it was possible to simply observe these publically available data without contacting the users directly for this research. However, there is still an ethical requirement to protect individually identifying information when the information is collected for research purposes. In this study, we used screen captures to record profile information, storing these pictures on a password-protected iPod Touch until the data of interest (age, race, and location information) could be entered into a database with no identifiers. Because we were recording only publically available data from user profiles without identifiers, the Institutional Review Board at Emory University considered the study to be research exempt from review.

More generally, using social networking apps for HIV prevention is likely a key strategy for future research [52-54] but comes with new ethical and methodological questions. Our study only sought to summarize the data publically available within these apps, but social media apps may themselves serve as an important public health communications tool. Recently, public health agencies have sought to partner with Grindr and use its built-in advertisements as a medium for disseminating prevention information and recruit MSM for research studies [52,53]. Future research might adapt our methodology further to establish a sampling frame and then use the density information to sample app users and contact them to either conduct a cross-sectional survey or recruit them into a follow-up study. At that time, one would have to develop mechanisms for consenting study participants, as well as a way to keep sensitive information, such as sex and drug use behavior, protected and ideally separate from any identifying online profile information.

### Limitations of the Current Work and Opportunities for Future Research

Piloting this methodology in Atlanta exposed other challenges as well. Atlanta is geographically large and contains both densely settled neighborhoods in the inner city along with a large amount of semi-urban and even rural areas with less dense populations. Atlanta also exhibits a large degree of geospatial segregation by race, both in the population overall [55] and in the relative measures of the distribution of social-network app users (Panel B in Figure 7). However, although the overall black population density is low in Midtown Atlanta [55,56], it still represented the area with the highest concentration of black users of the sex-seeking application (Figure 4). To obtain a picture of the distributions of both black and white app users, we therefore had to sample enough points with a sufficiently wide radius to cover the entire city. We also found the density of users to vary widely within the city, and we therefore had to adapt our sampling strategy. We chose to collect either the first 50 profiles and record the distance to the 50^th^ user, or to sample out to a 2-mile radius if there were less than 50 profiles observed in that area. In areas with large numbers of users, we had to collect data at more closely sampled points. For example, if there were 50 profiles within a half mile, we moved only that short distance before collecting more data. If there were only 13 users within the 2 mile radius, we moved the full 2 miles between sample points. This allowed us to cover the whole city, but despite collecting data at 79 points that represented an area equivalent to 882 square miles, there were still areas of the city where we did not directly sample any users.

This makes the choice of the smoothing parameter (radius) for the kernel smoothing algorithm important because it provides a balance between too much interpolation of data between sampling points and presuming that the data collected at a particular sampling point occur only at the point and do not represent an area defined by the radius of a circle based on the linear distance to the person whose profile is being observed. Using our sampling plan, we collected data from concentric circles with an average radius of 1.65 miles and then fit weighted kernel densities smoothed to 1 and 2 miles. Both of these smoothing parameters provided similar interpretations of density of black, white, and young black individuals, with the 1-mile radius leaving more areas of the city with no estimates for the density of app users. The 2-mile radius covers the whole city, but as a result it reduces the emphasis of several points which, when using a 1-mile radius are considered to have a particularly high density of black users.

Some questions remain about the precise interpretation of the density of social-network app users. For example, are users simply a subset of all MSM seeking sex on the Internet? Is the population that uses any one of these apps different by important characteristics (race, age, sex behavior with persons met through a social-network app or with sex partners generally) from the underlying population? Are persons who use specific services (eg, Adam-4-Adam, Jack’d, Grindr) different by one or more of these characteristics than those that use other online apps [47-49]? Future studies [54] will seek to quantify the density and characteristics of men who use each of these apps and compare the characteristics of men who use each of the apps exclusively, while also capturing information about men who use more than one service to describe whether their behaviors vary when using different services.

It would be useful to test the methodology in other cities with significant minority MSM populations (eg, Washington, DC, or Los Angeles, CA) and also to assess the utility of the method in less densely populated areas (eg, in rural areas of Georgia), to describe the extent to which the utility of the methods vary by characteristics of the geography of the region. We have already identified that Atlanta is a challenging place to conduct this kind of study because of its racial distribution, which was borne out in the social-network app user density data. In areas with sparse numbers of users, our adaptive sampling methodology, which sampled a maximum of 50 users or to a 2-mile radius, might help to stabilize density estimates, but this needs further testing. Additionally, although we averaged over day and time of sampling in our current analysis, the method could be refined to capture spatiotemporal trends in density. For example, it would be possible to select points to be sampled multiple times over a grid of specific times and days [14,15]. This modification could provide a clear description of how the user profile’s population density changes over the course of a week. This last component may identify trends in the spatial and temporal clustering of app users, for example on weekend nights, as compared to mid-day during the work week.

### Conclusions

We have found that it is possible to use a limited number of sample points to develop a geospatial density of men using a social-networking app to seek sex in the city of Atlanta. Such a density could serve as a sampling frame for future cross-sectional or longitudinal research. We also describe several methods to compare two densities with a goal of identifying areas with a high density of a particular subset of the population. We hope that this novel methodology and its further adaptations will prove useful to future research and prevention efforts that can be tailored to areas of the community where they will be most effective.

## Multimedia Appendix 1

Kernel density calculations and days/times of data collection.

## Abbreviations

HIV: human immunodeficiency virus
MSM: men who have sex with men
