# Supplementary material for: Using a Geolocation Social Networking Application to Calculate the Population Density of Sex-Seeking Gay Men for Research and Prevention Services
Source: J Med Internet Res. 2014 Nov 18;16(11):e249. doi: 10.2196/jmir.3523 (PMC4260063; doi:10.2196/jmir.3523)
Supplement: Supplementary file 1 [file jmir_v16i11e249_app1.pptx]

## Slide 1
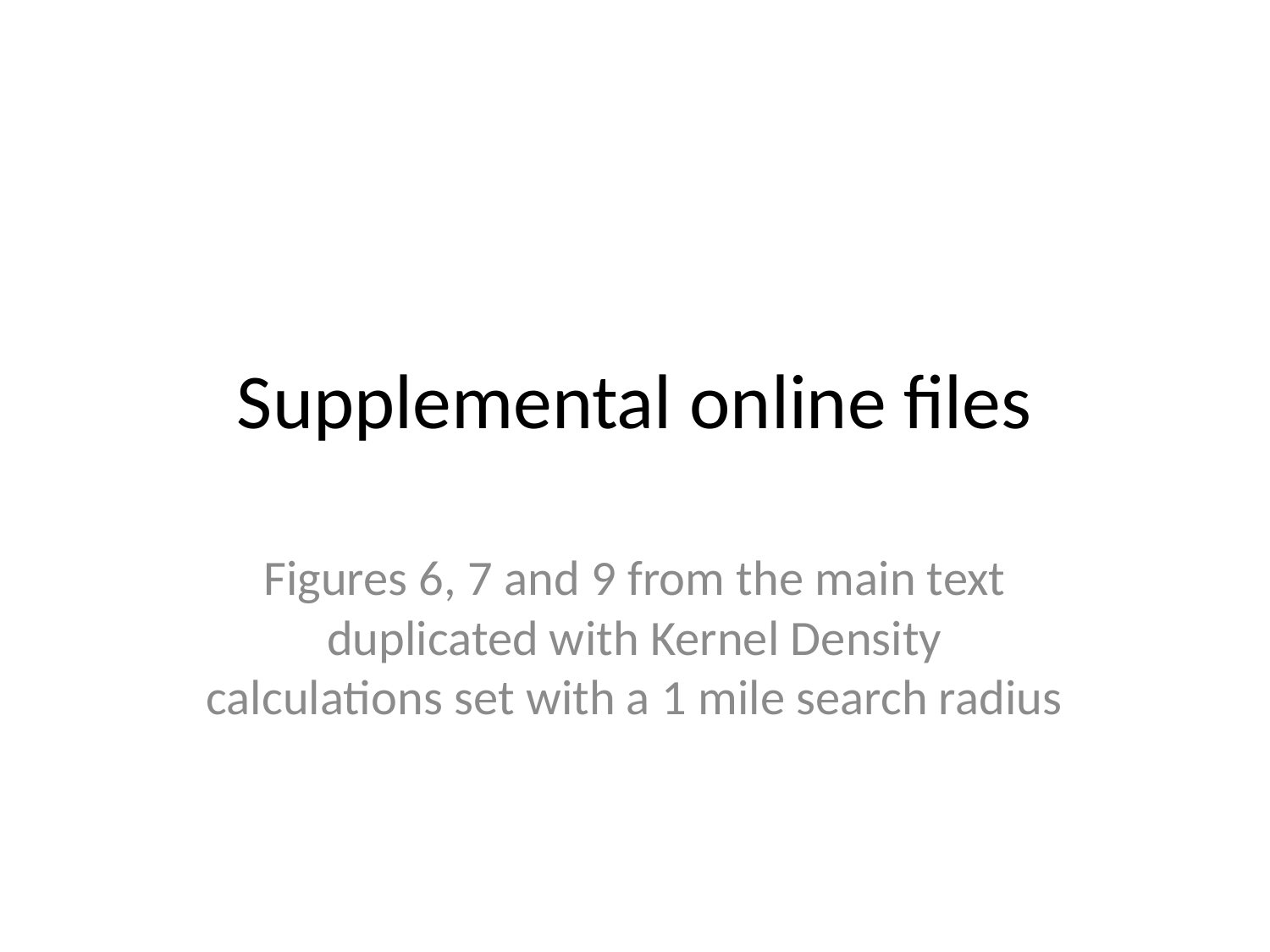

# Supplemental online files
Figures 6, 7 and 9 from the main text duplicated with Kernel Density calculations set with a 1 mile search radius

## Slide 2
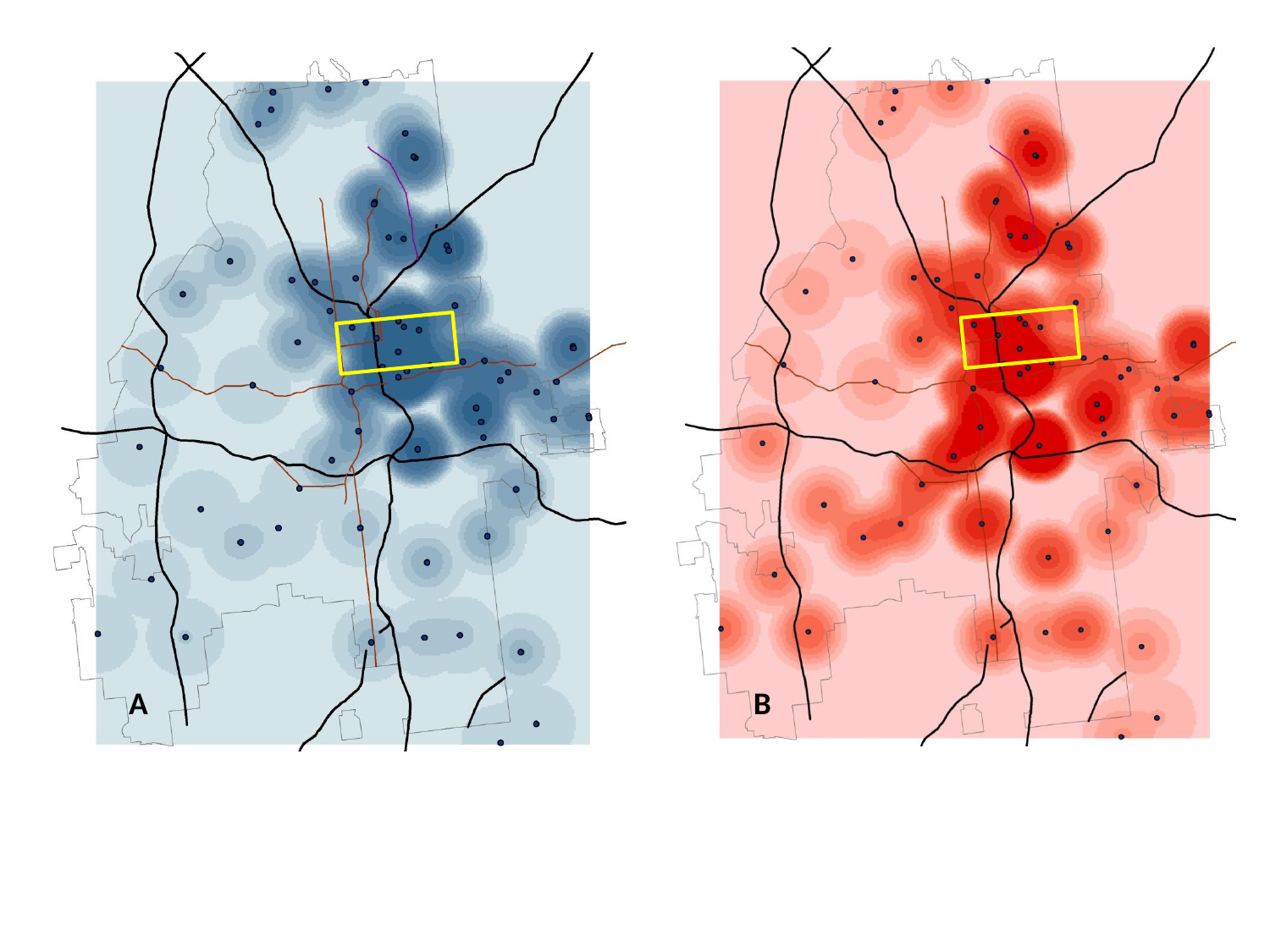

A
B

## Slide 3
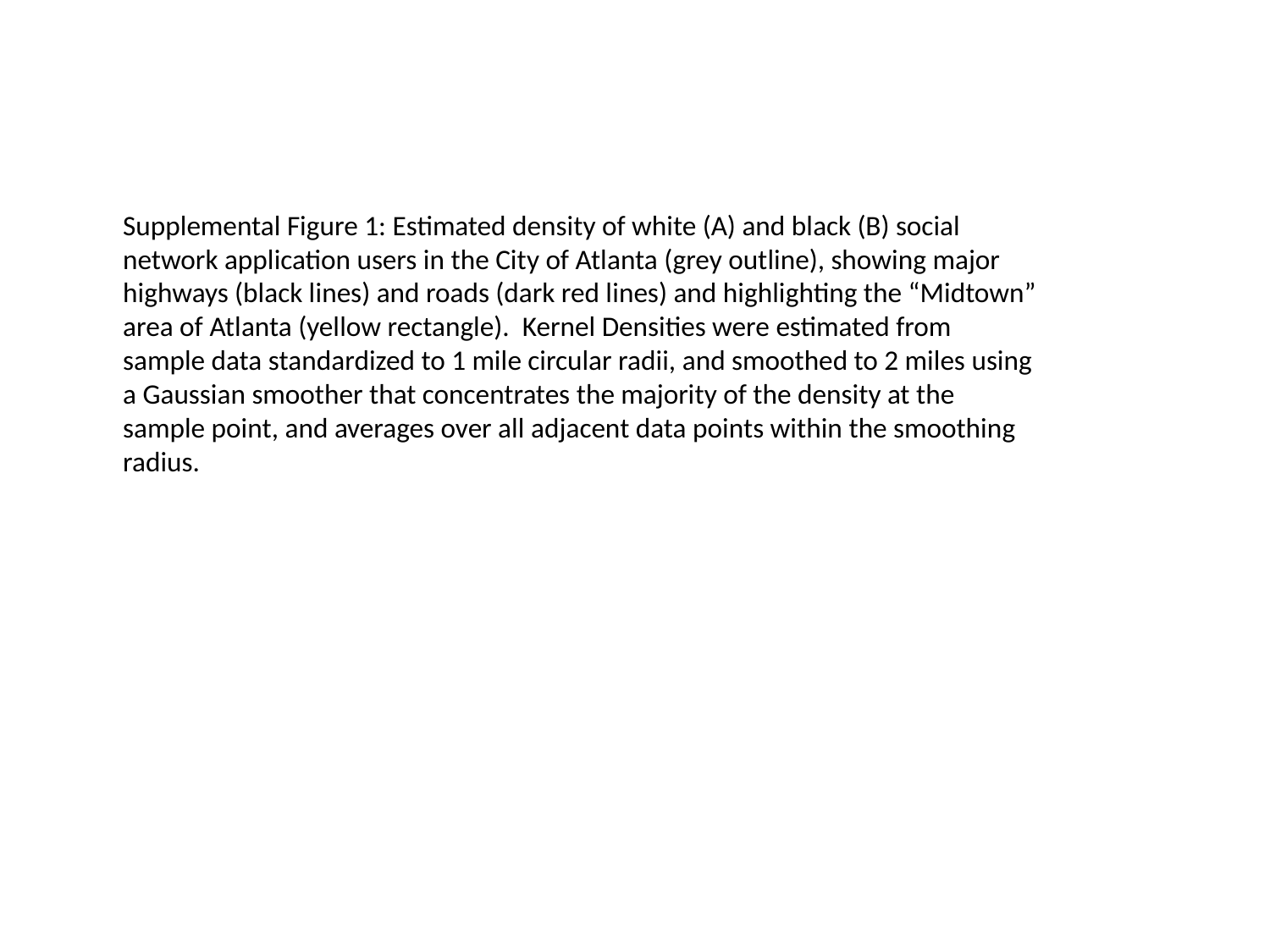

Supplemental Figure 1: Estimated density of white (A) and black (B) social network application users in the City of Atlanta (grey outline), showing major highways (black lines) and roads (dark red lines) and highlighting the “Midtown” area of Atlanta (yellow rectangle). Kernel Densities were estimated from sample data standardized to 1 mile circular radii, and smoothed to 2 miles using a Gaussian smoother that concentrates the majority of the density at the sample point, and averages over all adjacent data points within the smoothing radius.

## Slide 4
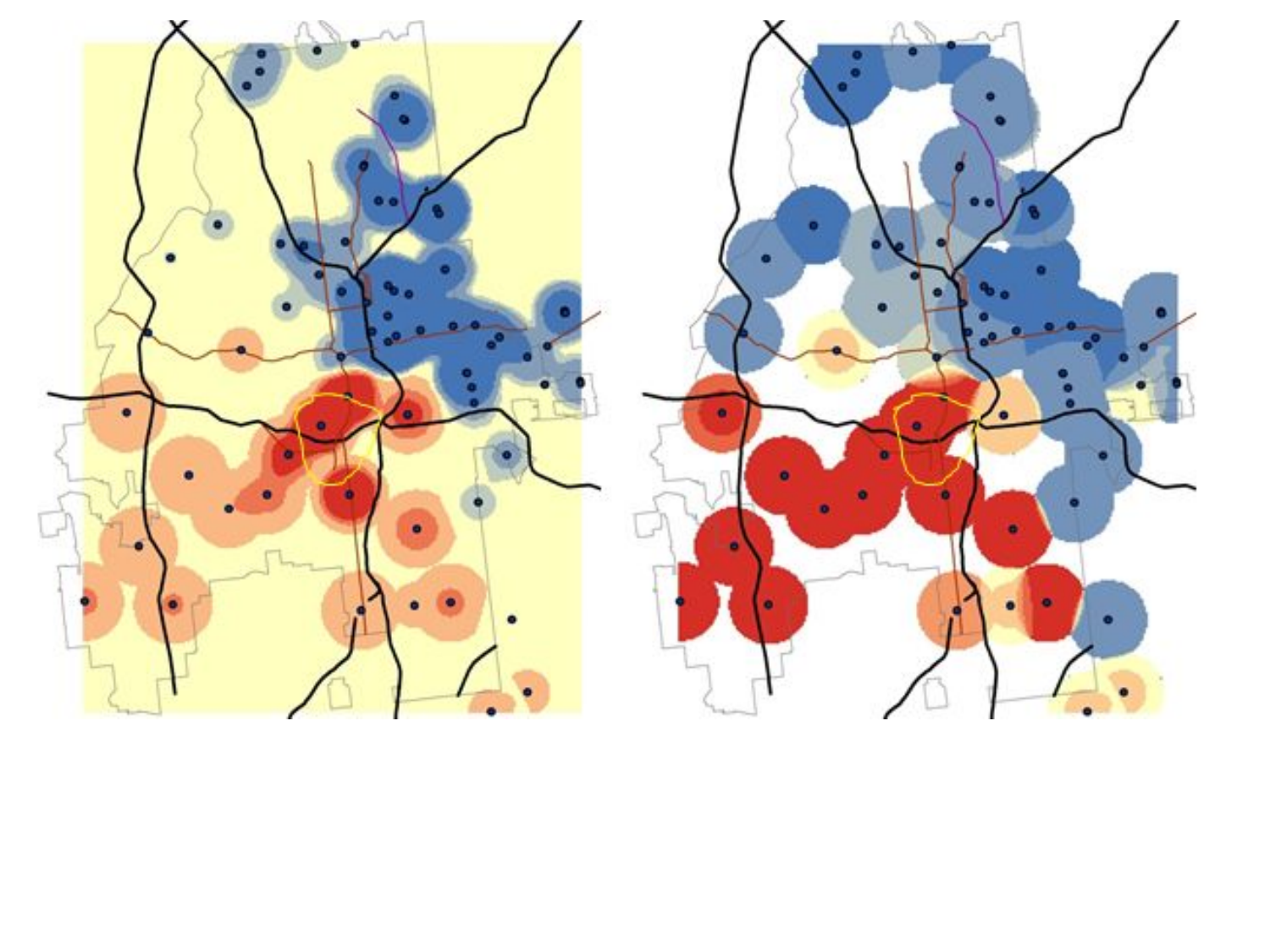

## Slide 5
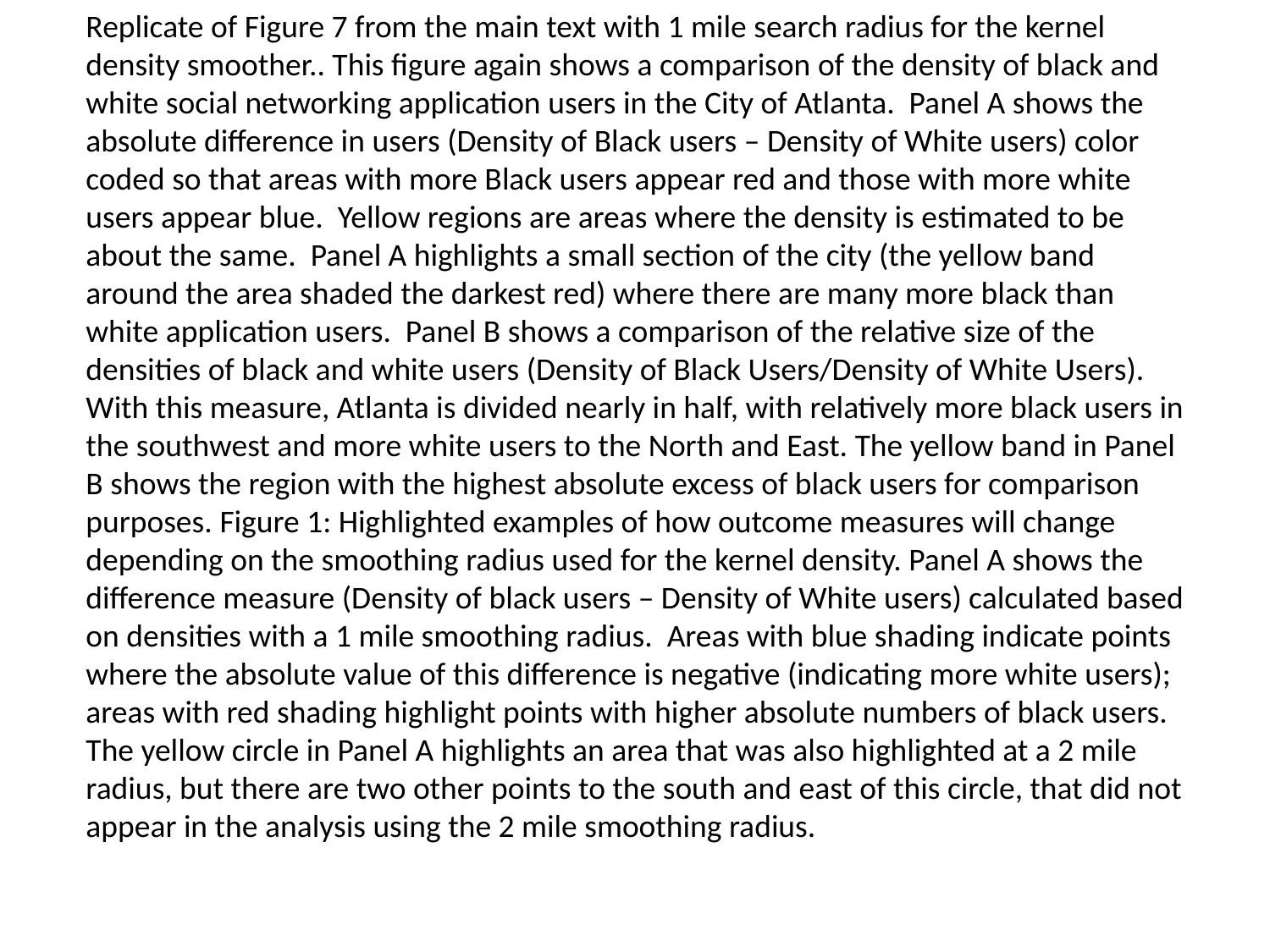

Replicate of Figure 7 from the main text with 1 mile search radius for the kernel density smoother.. This figure again shows a comparison of the density of black and white social networking application users in the City of Atlanta. Panel A shows the absolute difference in users (Density of Black users – Density of White users) color coded so that areas with more Black users appear red and those with more white users appear blue. Yellow regions are areas where the density is estimated to be about the same. Panel A highlights a small section of the city (the yellow band around the area shaded the darkest red) where there are many more black than white application users. Panel B shows a comparison of the relative size of the densities of black and white users (Density of Black Users/Density of White Users). With this measure, Atlanta is divided nearly in half, with relatively more black users in the southwest and more white users to the North and East. The yellow band in Panel B shows the region with the highest absolute excess of black users for comparison purposes. Figure 1: Highlighted examples of how outcome measures will change depending on the smoothing radius used for the kernel density. Panel A shows the difference measure (Density of black users – Density of White users) calculated based on densities with a 1 mile smoothing radius. Areas with blue shading indicate points where the absolute value of this difference is negative (indicating more white users); areas with red shading highlight points with higher absolute numbers of black users. The yellow circle in Panel A highlights an area that was also highlighted at a 2 mile radius, but there are two other points to the south and east of this circle, that did not appear in the analysis using the 2 mile smoothing radius.

## Slide 6
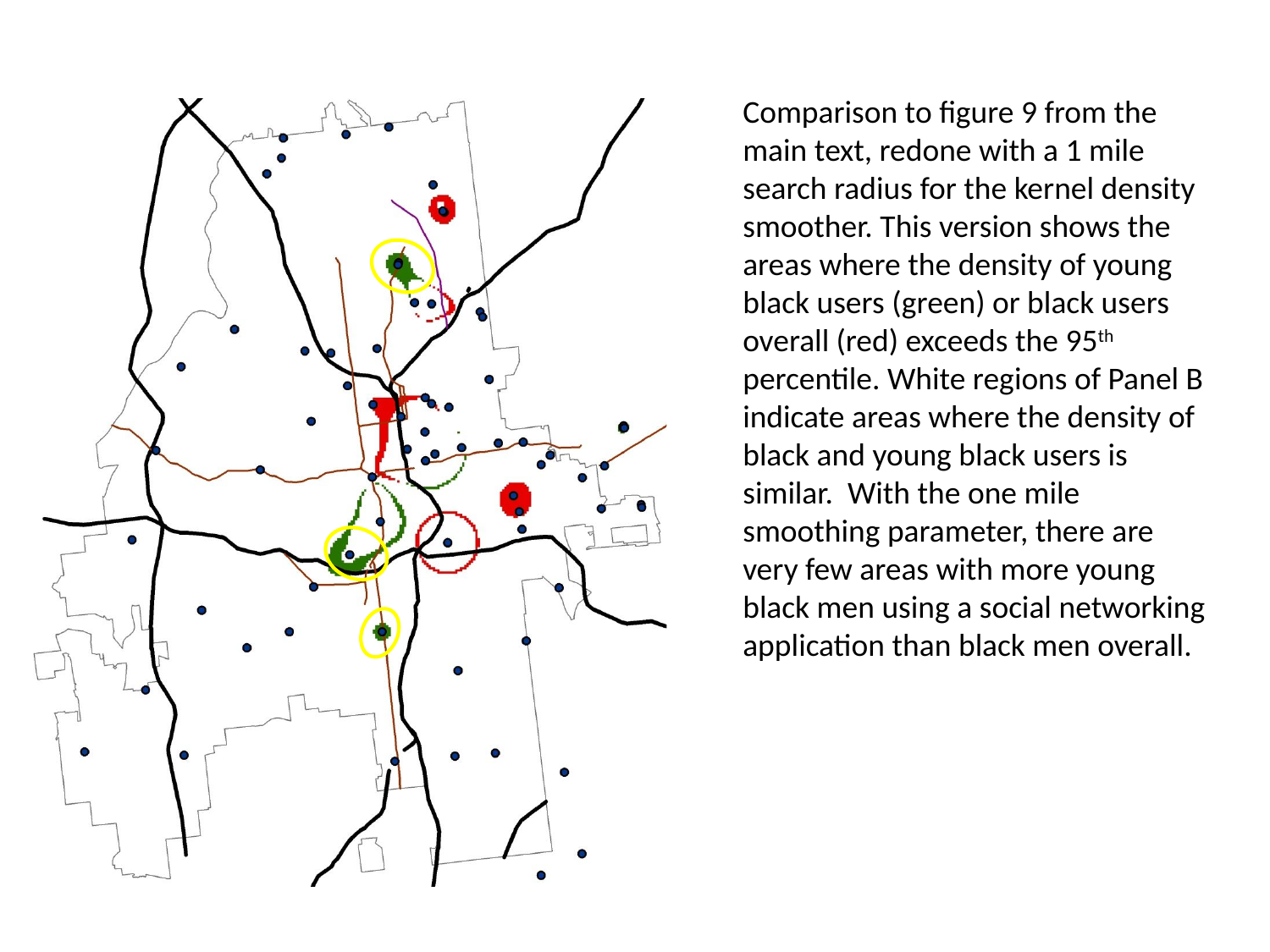

Comparison to figure 9 from the main text, redone with a 1 mile search radius for the kernel density smoother. This version shows the areas where the density of young black users (green) or black users overall (red) exceeds the 95th percentile. White regions of Panel B indicate areas where the density of black and young black users is similar. With the one mile smoothing parameter, there are very few areas with more young black men using a social networking application than black men overall.

## Slide 7
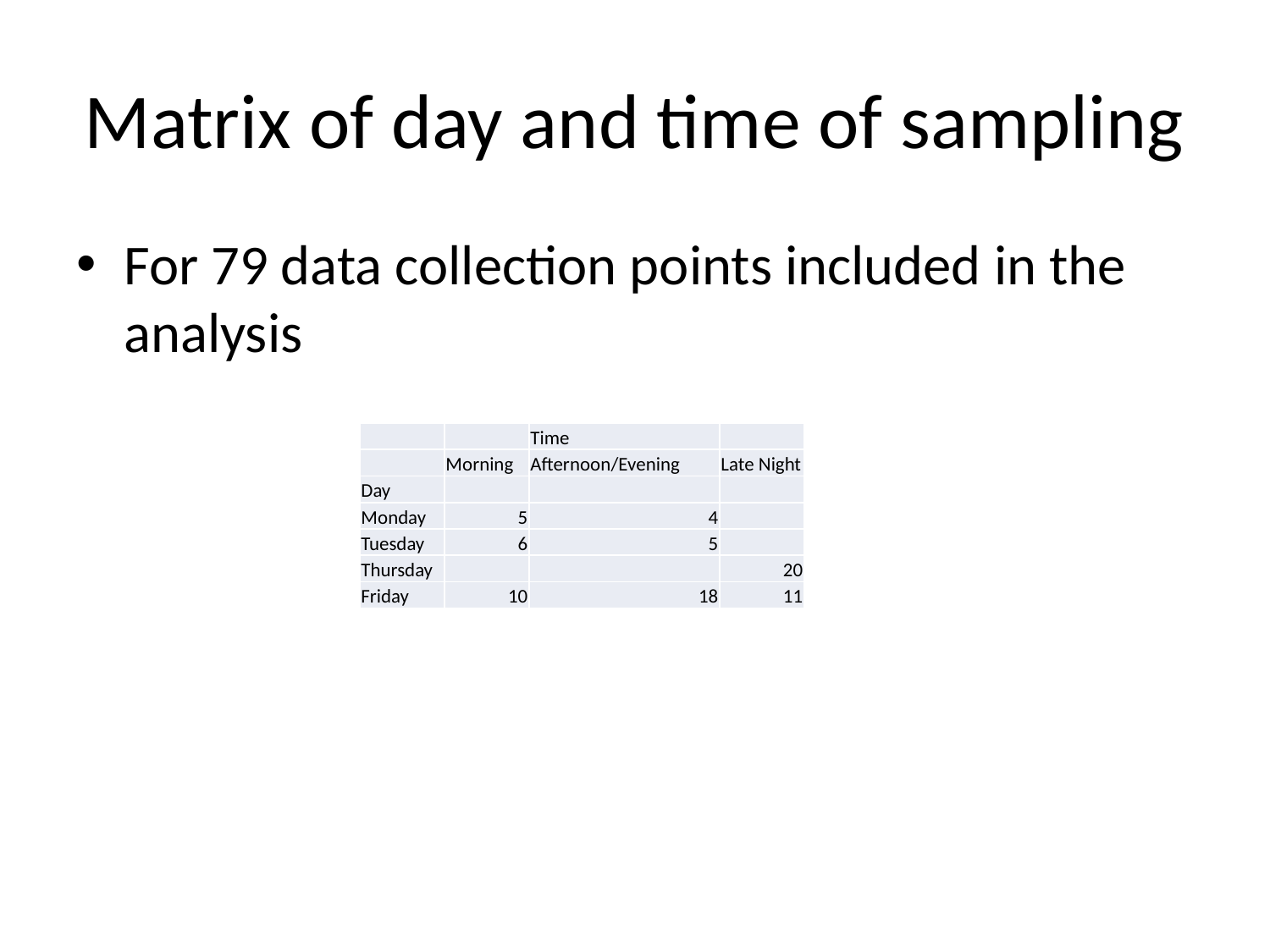

# Matrix of day and time of sampling
For 79 data collection points included in the analysis
| | | Time | |
| --- | --- | --- | --- |
| | Morning | Afternoon/Evening | Late Night |
| Day | | | |
| Monday | 5 | 4 | |
| Tuesday | 6 | 5 | |
| Thursday | | | 20 |
| Friday | 10 | 18 | 11 |
